# Effects of a job crafting intervention program on work engagement among Japanese employees: a pretest-posttest study

**DOI:** 10.1186/s40359-016-0157-9

**Published:** 2016-10-24

**Authors:** Asuka Sakuraya, Akihito Shimazu, Kotaro Imamura, Katsuyuki Namba, Norito Kawakami

**Affiliations:** 1 Department of Mental Health, Graduate School of Medicine, The University of Tokyo, 7-3-1, Hongo, Bunkyo-ku, Tokyo, 113-0033 Japan; 2 Chugai Pharmaceutical Company, 2-1-1, Nihonbashi muromachi, Chuo-ku, Tokyo, 103-8324 Japan

**Keywords:** Intervention, Job crafting, Psychological distress, Work engagement

## Abstract

**Background:**

Job crafting, an employee-initiated job design/redesign, has become important for employees’ well-being such as work engagement. This study examined the effectiveness of a newly developed job crafting intervention program on work engagement (as primary outcome), as well as job crafting and psychological distress (as secondary outcomes), using a pretest-posttest study design among Japanese employees.

**Methods:**

Participants were managers of a private company and a private psychiatric hospital in Japan. The job crafting intervention program consisted of two 120-min sessions with a two-week interval between them. Outcomes were assessed at baseline (Time 1), post-intervention (Time 2), and a one-month follow-up (Time 3). The mixed growth model analyses were conducted using time (Time 1, Time 2, and Time 3) as an indicator of intervention effect. Effect sizes were calculated using Cohen’s d.

**Results:**

The program showed a significant positive effect on work engagement (t = 2.20, *p* = 0.03) in the mixed growth model analyses, but with only small effect sizes (Cohen’s d = 0.33 at Time 2 and 0.26 at Time 3). The program also significantly improved job crafting (t = 2.36, *p* = 0.02: Cohen’s d = 0.36 at Time 2 and 0.47 at Time 3) and reduced psychological distress (t = −2.06, *p* = 0.04: Cohen’s d = −0.15 at Time 2 and −0.31 at Time 3).

**Conclusions:**

The study indicated that the newly developed job crafting intervention program was effective in increasing work engagement, as well as in improving job crafting and decreasing psychological distress, among Japanese managers.

**Trial registration:**

UMIN Clinical Trials Registry UMIN000024062. Retrospectively registered 15 September 2016.

## Background

Today’s organizations require their employees to be motivated, proactive, responsible, and involved. In other words, modern work organizations need engaged employees [1, 2].

**Table 1:** Characteristics of participants from two work places in Japan (*N* = 50)

	A manufacturing company (*n* = 36)				A private psychiatric hospital (*n* = 14)				Total (*N* = 50)			
	n	(%)	Mean	SD	n	(%)	Mean	SD	n	(%)	Mean	SD
Gender												
Male	34	(94.4)			8	(57.1)			42	(84.0)		
Female	2	(5.6)			6	(42.9)			8	(16.0)		
Age (Years)			49.4	6.2			38.4	8.7			46.3	8.5
Educational attainment												
University or higher	13	(36.1)			8	(57.1)			21	(42.0)		
High school or some college	23	(63.9)			6	(42.9)			29	(58.0)		
Marital status												
Married	29	(80.6)			11	(78.6)			40	(80.0)		
Not married	7	(19.4)			3	(21.4)			10	(20.0)		
Working hours per week^a^			45.4	6.0			47.9	5.5			45.9	5.9

^a^The mean and standard deviation of working hours per week was calculated for 33 and 9 participants from a manufacturing company and a private psychiatric hospital, respectively, who had no missing response on this variable

**Table 2:** Means (SDs) of outcome variables at baseline, post-intervention and one-month follow-up^a^

	Range	Time 1 (*N* = 50)		Time 2 (*N* = 44)		Time 3 (*N* = 42)	
Work engagement	0–6	2.61	(0.74)	2.74	(0.83)	2.79	(0.91)
Job crafting (total)	1–7	4.73	(0.83)	4.91	(0.95)	4.98	(0.80)
Task crafting	1–7	4.93	(0.93)	5.06	(0.98)	5.09	(0.79)
Relational crafting	1–7	4.89	(0.97)	5.02	(1.02)	5.06	(0.94)
Cognitive crafting	1–7	4.38	(1.10)	4.66	(1.17)	4.80	(0.95)
Psychological distress	1–4	2.02	(0.69)	1.92	(0.72)	1.82	(0.62)

^a^Time 1, baseline; Time 2, post intervention; Time 3, one-month follow-up

**Table 3:** Effects of a job crafting intervention program on work engagement, job crafting, and psychological distress in the mixed growth model analyses for the whole sample (*N* = 50)

	Estimates of fixed effects (Crude)						Estimates of fixed effects (Adjusted)^a^			
	Effect	*t*	*p*	95 % CI		Effect	*t*	*p*	95 % CI	
				lower	upper				lower	upper
Work engagement	0.11	2.23	0.03	0.01	0.21	0.11	2.20	0.03	0.01	0.21
Job crafting	0.13	2.37	0.02	0.02	0.25	0.13	2.36	0.02	0.02	0.25
Task crafting	0.08	1.28	0.20	−0.04	0.20	0.08	1.25	0.21	−0.05	0.20
Relational crafting	0.09	1.28	0.20	−0.05	0.23	0.09	1.28	0.20	−0.05	0.23
Cognitive crafting	0.23	2.75	0.01	0.06	0.40	0.23	2.76	0.01	0.07	0.40
Psychological distress	−0.08	−2.07	0.04	−0.15	−0.003	−0.08	−2.06	0.04	−0.15	−0.003
	Cohen’s *d*									
	T2-T1^b^	95 % CI			T3-T1^c^	95 % CI		T3-T2^d^	95 % CI	
		lower	upper			lower	upper		lower	upper
Work engagement	0.33	0.03	0.62		0.26	−0.04	0.56	0.10	−0.21	0.42
Job crafting	0.36	0.06	0.65		0.47	0.17	0.77	0.03	−0.28	0.35
Task crafting	0.15	−0.15	0.44		0.32	0.02	0.62	0.00	−0.31	0.31
Relational crafting	0.23	−0.06	0.53		0.20	−0.10	0.50	−0.04	−0.35	0.28
Cognitive crafting	0.34	0.04	0.64		0.56	0.26	0.86	0.12	−0.19	0.44
Psychological distress	−0.15	−0.44	0.15		−0.31	−0.62	−0.01	−0.23	−0.54	0.09

*CI* confidence interval, *T1* baseline, *T2* post intervention, *T3* one-month follow-up

^a^Adjusted for gender, age, marital status, and educational attainment

^b^Cohen’s *d* between T1 and T2 were based on the score of the respondents who completed baseline survey and post intervention survey (*N* = 44)

^c^Cohen’s *d* between T1 and T3 were based on the score of the respondents who completed baseline survey and one-month follow-up survey (*N* = 42)

^d^Cohen’s *d* between T2 and T3 were based on the score of the respondents who completed post intervention survey and one-month follow-up survey (*N* = 39)

Work engagement is defined as an active, positive, work-related state of mind characterized by vigor, dedication, and absorption [3, 4]. According to empirical studies with longitudinal designs [5–8], engaged workers show low psychological distress, few physical complaints, and high employee performance. In addition, a meta-analytic study [9] showed that work engagement is negatively related to turnover intention and positively related to employee performance and organizational commitment.

The number of intervention studies aimed at improving work engagement is increasing. Although seven randomized controlled trials (RCTs) have been conducted since 2012, only two studies showed an improvement of work engagement at follow-up.

For instance, Coffeng et al. (2014) conducted an intervention to change the physical work environment (e.g., creating a coffee corner) and found a significant improvement of absorption, a component of work engagement [10]. However, they did not find a significant improvement in overall work engagement (i.e., total score of Utrecht Work Engagement scale) [10]. Another example is the study of Imamura and his colleagues (2015), in which internet cognitive behavioral therapy (iCBT) was employed. Although they showed a significant improvement of work engagement at six-month follow-up, its effect size was small (0.16) [11].

One possible reason why previous studies failed to find an intervention effect on work engagement (or found only a small one) is that they did not employ strategies to simultaneously improve the two resources of work engagement – that is, job resources and personal resources [1, 8]. Specifically, those studies employed programs changing the physical work environment [10], programs improving psychological resources through cognitive behavioral therapy [11], career management skill workshops [12], mindfulness training sessions [13, 14], or lifestyle change programs [15, 16]. These programs only focused on one or the other of the two resources. Hence, if a program were to focus on both job and personal resources at the same time, a better intervention effect might be obtained.

Job resources include physical, social, or organizational aspects of the job that help individuals to achieve their working goals; stimulate personal growth, learning, and development; or reduce job demands or associated physical or psychological costs, such as support from colleagues and opportunity for development [17]. Personal resources are positive self-evaluations that are linked to resiliency and refer to individuals’ sense of their ability to successfully control and have an impact on their environment, such as self-efficacy and optimism characteristics [8, 18, 19].

Job crafting – that is, employee-initiated design/redesign of work characteristics – can be conceptualized as a personal resource. According to Wrzesniewski and Dutton (2001), job crafting is defined as “the physical and cognitive change individuals make in the task or relational boundaries of their work” [20]. Job crafting consists of the following three components: changing the job’s boundaries (task crafting), changing the relational boundaries (relational crafting), and changing the cognitive task boundaries (cognitive crafting) [20, 21]. Because task crafting or relational crafting can lead to designing and improving one’s work and social environment in the workplace [20, 22], an intervention program that focuses on these components of job crafting may be effective for increasing job resources as well. Indeed, previous observational studies have reported that job crafting was positively associated with job resources and work engagement [23–25] and negatively associated with burnout, an opposite aspect of work engagement [23]. Therefore, a job crafting intervention program may be effective to increase work engagement.

To date, there have been only three non-randomized controlled trials of job crafting program, which composed of two or three group sessions [26–28]. For instance, Wingerden et al. (2016) showed the significant effect of the intervention on work engagement, psychological capital, and in-role performance [28]. However, the other two studies found no significant intervention effects on job resources (opportunities for development and leader–member exchange), personal resource (self-efficacy), positive/negative affect at work [27], or work engagement, performance [26]. Accordingly, we may say that the effect of job crafting intervention on work-related outcome is unclear. In addition, all of the previous interventions focused only on changing job resources or demands, and were not fully based on the original concept of job crafting by Wrzesniewski and Dutton [20].

The aim of this study was to investigate the effectiveness of a newly developed job crafting intervention program on work engagement among Japanese employees with a pretest-posttest study design. We expect that the scores of job crafting (for manipulation check) and work engagement (primary outcome) will increase at Time 2 (post-intervention) and at Time 3 (one-month follow-up) than compared to Time 1 (baseline). In addition, the score of psychological distress (secondary outcome) will decrease at Time 2 and Time 3 compared to Time 1. The strong point of this program is that it focuses on three types of job crafting (i.e., task, relation, and cognition) based on the concept of Wrzesniewski and Dutton [20], which may be useful to increase work engagement.

## Methods

### Study design

A pre- and post-intervention study was conducted. There was no control group because it was difficult to set due to the condition of the participant organizations. Therefore, we put this study as a pilot study to investigate the effectiveness of our job crafting intervention preliminarily. The study protocol was registered retrospectively at the UMIN Clinical Trials Registry (UMIN-CTR) (ID = UMIN000024062). The ethics review board of The University of Tokyo Graduate School of Medicine/Faculty of Medicine approved the procedures before the start of the study (the reference number: 10749). This manuscript was reported according to the TREND statement checklist [29].

### Participants

This study was carried out from August to November 2015 at two private companies in Japan. We recruited all full-time managers in a manufacturing company (*n* = 54) and managers who belonged to seven selected departments at a private psychiatric hospital (*n* = 25). Participants were approached by a contact person in their own company or hospital using an e-mail invitation or a poster. The inclusion criterion for participants was regular (full time) employment; workers with non- regular (part time) employment and reemployment in a temporary position were excluded.

### Interventions

The intervention program, which was based on the job crafting theory of Wrzesniewski and Dutton (2001) [20], was developed through literature review [20–25], discussion with occupational health professionals, and interview with employees on how they craft their own job in their working lives, such as changing the relationships with others or reframing the significance of their work. One of the unique features of the program, compared to previous job crafting interventions [26–28], was that it contains three aspects of job crafting (i.e., task, human relation, and cognition), which may be important to work engagement. The program consisted of two 120-min sessions with a two-week interval between them and was conducted by one researcher (the first author) and one clinical psychologist. The two training sessions were held in a room at the worksite in a group setting with 9–13 participants during working hours in the company, separately for department 1 (first session *n* = 11, second session *n* = 9), department 2 (*n* = 12 and 13, respectively), and department 3 (*n* = 12 and 12, respectively). The sessions were conducted outside of working hours in the hospital (*n* = 13 and 10, respectively). In the first session, following the introduction of the idea of job crafting including task, relational or cognitive crafting, by the researcher, participants learned the concept of job crafting from a case study (30 min), shared their personal crafting stories in their own working lives (30 min), and made their own individual job crafting plans (task crafting, relational crafting, and cognitive crafting) for the next two weeks (30 min). They were also provided with a homework booklet for a job crafting exercise. During the two weeks between the first and second sessions, they were encouraged to implement their job crafting plans. In the second session, each participant reviewed his/her own job crafting plan individually (15 min); the participants then shared their reflections as a group (30 min), discussed what job crafting would be feasible and sustainable to practice (30 min), and, finally, made a modified job crafting plan (30 min). Participants who could not attend first session were distributed the material of the session and asked to make their job crafting plan and practice it before they attend the second session. Thus, they could attend the second session. There was no incentive offered for participation in the program.

### Outcomes

All data were collected using a web-based self-report questionnaire at baseline, post-intervention, and one-month follow-up.

### Primary outcome

#### Work engagement

Work engagement was assessed using the Japanese version of the Utrecht Work Engagement Scale (UWES), which has been reported to be reliable and valid [30]. It comprises 9 items assessing vigor (3 items; e.g., ‘At my work, I feel bursting with energy.’), dedication (3 items; e.g., ‘My job inspires me.’), and absorption (3 items; e.g., ‘I get carried away when I am working.’). All items were rated on a seven-point Likert scale ranging from 0 (never) to 6 (always), and the total score for each subscale was divided by the number of items to get an average score.

### Secondary outcomes

#### Psychological distress

Psychological distress was measured using the Brief Job Stress Questionnaire (BJSQ) [31], comprising 15 items assessing irritation (3 items; e.g., ‘I feel anger’), fatigue (3 items; e.g., ‘I feel very tired’), anxiety (3 items; e.g., ’I feel uneasy’), and depression (6 items; e.g., ‘I feel depressed’). All items were measured on a four-point Likert scale ranging from 1 (never) to 4 (almost always), and the total score was calculated by dividing the sum of item scores by the number of items in the present study.

### Manipulation check of the intervention

#### Job crafting

Job crafting was assessed using a scale developed by Sekiguchi and his colleagues [21] based on the conceptualization by Wrzesniewski and Dutton (2001) [20], which has been reported to be reliable and valid [21]. It comprises 12 items assessing three subscales: task crafting (4 items; e.g., ‘Add or reduce tasks so that my job can be performed more smoothly’), relational crafting (4 items; e.g., ‘Actively interact with people through my job’), and cognitive crafting (4 items; e.g., ‘Reframe my job as significant and meaningful’). All items were measured on a seven-point Likert scale ranging from 1 (strongly disagree) to 7 (strongly agree), and the total score, as well as each subscale score, was calculated by dividing the sum of item scores by the number of the items.

### Demographic characteristic

Demographic variables included age, gender (0 = male, 1 = female), marital status (0 = married, 1 = not married), educational attainment (0 = university or higher, 1 = high school or some college), and working hours per week were collected. These variables could be possibly confounded in the association between work-related variables and employee well-being [1, 21, 23, 32, 33]. A close investigation of the responses to work hours revealed that some respondents reported less than 35 h per week, while all respondents were employed full-time. These responses were coded as missing values.

### Sample size

The estimated sample size was 66 participants to detect an effect size (Cohen’s *d*) of 0.35 or greater for work engagement, at an alpha error rate of 0.05 (two-tailed) and a beta error rate of 0.20 using the G*Power 3 program [34, 35]. No previous studies have reported an effect size of the job crafting intervention program for work engagement. However, referring to a previous study [27] that showed the effect size (Cohen’s *d*) for self-efficacy and job-related affective well-being, it seemed reasonable to set 0.35 as an expected effect size in our job crafting intervention program.

### Statistical methods

The analysis was performed at the individual level. A mixed model for repeated measures conditional growth model analysis over time (baseline, post-intervention, and one-month follow-up) was conducted to test the effect of the intervention. The mixed model is useful to understand changes in human behavior over time because of higher-level clustering unit (i.e., time) [36, 37]. First, we applied several mixed models to the data: random intercept and random slope; random intercept only; and random slope only. A converged model that showed the smallest AIC (Akaike Information Criterion), an indicator of goodness of fit of the model, was selected. If these mixed models did not converge, a fixed model was used. The linear mixed model in SPSS Statistics 22.0 (SPSS Inc, Chicago, IL) was used. Also, the effect sizes and the 95 % confidence intervals (95 % CIs) were calculated using Cohen’s *d* only among those who completed the questionnaire at post-intervention or follow-up, although the effect sizes may be biased due to drop-outs [38, 39]. Values of 0.2, 0.5, and 0.8 are generally interpreted as being suggestive of small, medium, and large effects, respectively [39].

## Results

### Participant flowchart

**Fig. 1:**
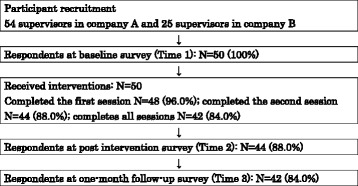
Participants flow diagram. Participants were recruited from one company and one hospital in Japan; all senior staff in company A (*n* = 54) and supervisors who belonged to seven selected departments at the private psychiatric hospital B (*n* = 25). 50 employees (63.3 %) participated in this study

The participant flowchart is shown in Fig. 1. A total of 50 employees participated in this study and completed the baseline survey (Time 1). Of those, 44 participants (88 %) and 42 participants (84 %) completed the post-intervention survey (Time 2) and the one-month follow-up survey (Time 3), respectively. For the process evaluation, 48 (96 %) completed the first session, 44 (88 %) completed the second session, and 42 (84 %) completed all session.

### Baseline data

Demographic characteristics of participants in subgroups and total are presented in Table 1. In the whole sample, most were males, middle-aged and married; more than half received high school or some college education. Working hours per week were reported from 42 participants in the total, with the average of 45.9 h per week, ranging from 35 to 60. Proportion of males and average age were significantly greater for participants from the manufacturing company than those from the private psychiatric hospital (*p* < .05 and *p* < .01, respectively); no significant difference was observed in other variables between the two groups (*p* > .05). There were also no significant differences in demographic characteristics at baseline between those who completed surveys (*n* = 39) and those who did not complete all surveys (*n* = 11) (*p* > .05).

### Means (SDs) of outcome variables at baseline, post-intervention and one-month follow-up

Table 2 shows the means and standard deviations of the outcome variables at baseline (Time 1), post-intervention (Time 2), and one-month follow-up (Time 3). The means of work engagement, job crafting (total), and all subscales of job crafting tended to increase over time constantly, whereas that of psychological distress tended to decrease over time.

### Effects on outcome variables

#### Effect on work engagement

Table 3 shows the estimated effects of the job crafting intervention program on the outcome variables on the basis of the mixed model analyses. Any of the models including random effects did not converge. The results from the model including only fixed effects were reported here. The job crafting intervention program showed a significantly positive effect on work engagement before and after adjustment of covariates (*t* = 2.23, *p* = 0.03; *t* = 2.20, *p* = 0.03). The effect sizes were small and significant at post-intervention (Time 2): Cohen’s *d* = 0.33 (95 % CI, 0.03 to 0.62), but not significant at one-month follow-up (Time 3) Cohen’s *d* = 0.26 (95 % CI, −0.04 to 0.56).

#### Effect on psychological distress

The program showed a significantly favorable effect on psychological distress before and after adjusting the covariates (*t* = −2.07, *p* = 0.04; *t* = −2.06, *p* = 0.04). The effect sizes were small and non-significant at post-intervention (Time 2): Cohen’s *d* = −0.15 (95 % CI, −0.44 to 0.15), but significant at one-month follow-up (Time 3): Cohen’s *d* = −0.31 (95 % CI, −0.62 to −0.01).

#### Effect on job crafting

The program showed a significantly positive effect on job crafting before and after adjusting the covariates (*t* = 2.37, *p* = 0.02; *t* = 2.36, *p* = 0.02), with small effect sizes at post-intervention (Time 2) (Cohen’s *d* = 0.36, 95 % CI, 0.06 to 0.65) and at one-month follow-up (Time 3) (Cohen’s *d* = 0.47, 95 % CI, 0.17 to 0.77). For the subscales, the program also showed a significant effect on cognitive crafting before and after adjustment of covariates (*t* = 2.75, *p* = 0.01; *t* = 2.76, *p* = 0.01), with small effect sizes at post-intervention (Time 2) (Cohen’s *d* = 0.34, 95 % CI, 0.04 to 0.64) and at one-month follow-up (Time 3) (Cohen’s *d* = 0.56, 95 % CI, 0.26 to 0.86). On the other hand, the program did not show significant effects on task crafting or relational crafting (*p* > .05); in addition, effect sizes were small and not significant, excepting for task crafting at one-month follow-up (Time 3) (Cohen’s *d* = 0.32, 95 % CI, 0.02 to 0.62).

## Discussion

The present pretest-posttest study examined the effects of a newly developed job crafting intervention program on work engagement and other outcomes (i.e., job crafting and psychological distress) among Japanese employees. The novel aspect of the current program is that it contains three factors of job crafting, such as task, relational, and cognitive crafting, which focuses on improving two preceding factors of work engagement (i.e., personal and job resources) [20]. The study found a significantly favorable effect of the program on work engagement, total and cognitive job crafting, and psychological distress at one-month follow-up.

### Effect on work engagement

The program significantly increased work engagement. Only two previous studies [26, 28] investigated the effect of a job crafting program on work engagement, and these results were inconsistent, probably because their program did not include the full components of job crafting (i.e., lacking a focus on cognitive crafting). The present result was also in line with previous findings from observational studies of positive associations between job crafting and work engagement [23–25].

The present study also found that the program effectively improved job crafting of participants, and particularly cognitive job crafting. We can assume that the current program promoted work engagement through job crafting, especially cognitive crafting. According to the extended Job Demands-Resources model [17], work engagement is improved by job resources and personal resources [8, 9, 17–19]. A possible reason why cognitive crafting was more improved than other types of job crafting is that cognitive crafting may be easily improved since it requires only changing their cognition about their work; on the other hand, task or relational crafting requires them to change their behaviors and actions at work [20, 21]. Therefore, it may be important for employers to provide their employees with opportunities to craft tasks or relations, which would encourage employees to change their own behaviors and actions at work.

However, the effect sizes for work engagement were small, and the favorable effect at post-intervention survey (Time 2) disappeared at one-month follow-up (Time 3). One possible reason for this is that the program did not improve all aspects of job crafting. This speculation seems to be supported by the results that the program did not significantly improve task crafting and relational crafting. As mentioned above, compared to cognitive crafting, it seems difficult for participants to acquire skills to improve task and relational crafting, because the latter focus on changing the actual job and relational boundaries. The program may have needed a session focusing on enhancing the application of the acquired skills in the participants’ daily working lives. For instance, in a previous job crafting training program for police officers [27], participants had opportunities to practice job crafting for four weeks after one day of training and received a half-day session to wrap up. While the present program had the discussion to share and analyze personal crafting (30 min) after two-week practice, the time for discussion may be too short for participants to make a detailed scenario of how to apply the acquired skills in their working life. Future programs may need to include longer time periods for such group discussions to enhance task and relational crafting.

In addition, the interval period between the sessions may have been too short for participants to practice their job crafting plans. The current job crafting intervention program had a two-week interval between the sessions, which is shorter than a previous job crafting program with a four-week interval [27]. In our results, cognitive crafting significantly improved, but task and relational crafting did not. It might take shorter time for participants to craft their own jobs cognitively, but take longer time to craft their tasks and relationship at work. A longer interval period may be needed to develop favorable effects on work engagement by improving task and relational crafting.

### Effect on psychological distress

The program significantly decreased the psychological distress of participants (*p* = 0.04). This result is in accordance with previous findings from observational studies of the association of job crafting with negative psychological outcomes [23]. A previous intervention study also reported a significant effect of a job crafting intervention on improving negative affect at work in a pre-post comparison within an intervention group only [27]. The present result supports this finding, suggesting the effectiveness of a job crafting intervention on mitigating psychological distress. This favorable effect on psychological distress may also be mediated by job crafting. The current job crafting program may have increased participants’ job resources, which contributed to decreasing their psychological distress. In addition, their acquired job crafting skills (especially cognitive crafting) could act as coping strategies in managing stressful working conditions. Specifically, reframing one’s own job as significant and meaningful (i.e., cognitive crafting) can function as positive reappraisal, a form of coping strategy. Given that positive reappraisal (i.e., reinterpretation of the situation to either reduce the severity of the negative response or exchange the negative attitude for a more positive attitude) is negatively correlated with negative psychological outcomes [40], cognitive crafting may have favorable effects on decreasing psychological distress in the form of positive reappraisal. However, again, it should be noted that the effect sizes were small and significant only at one-month follow-up (Time 3), but not significant at post-intervention (Time 2). Because it may take some time to transfer the acquired skills (especially task crafting and relational crafting) to real life, longer-term observation can clarify such intervention effects [41].

### Theoretical contribution and future directions

Our findings contribute to future research not only practical but also theoretical points of view. First, our job crafting program was found to have positive effects on cognitive crafting and work engagement. Our findings empirically support a theoretical prediction of job crafting, at least cognitive crafting, could improve work engagement. Because work engagement is a work-related affective cognitive state [3, 42], it may be influenced more by the cognitive type of job crafting. Future research needs to clarify the process of how cognitive crafting leads to improved work engagement. Second, task and relational crafting were not much changed in the present intervention study,　although our job crafting program targeted all three aspects of job crafting. Other conditions than an individual-focused training may be needed to improve these types of crafting. According to the concept model of job crafting by Wrzesniewski and Dutton (2001), employees may need prerequisites (e.g., need for control over job and work meaning, positive self-image, and human connection with others) to develop task and relational crafting [20]. Exploring factors and conditions for different types of job crafting may be important for further theoretical development of job crafting.

### Limitations

This study has several limitations. First, the current study did not have a control group. Thus, it is unclear whether the change of work engagement and other outcomes is caused by the intervention program or due to other reasons, such as a natural course. The effect of the program on these outcomes should be examined by a randomized control trial (RCT) design.

Second, the sample size of this study (*N* = 50) was modest, compared to the planned number of 66 to detect an effect size of 0.35 or greater for work engagement. Thus, the study had lower statistical power due to the small sample size than that with the planned sample size.

Third, the sample consisted of only senior staff in a company and supervisors in a hospital, who might have more job discretion compared to other employees. Given a positive association between job autonomy and job crafting [21], these respondents could more easily transfer their acquired skills to a real-life setting. Therefore, the generalization of the current findings is limited. A further intervention study should be conducted in other occupational groups.

Fourth, the follow-up period was relatively short compared with previous studies that focused on work engagement [11]. Because it may take time to transfer the acquired skills (especially task crafting and relational crafting) to a real-life setting, a longer-term observation is needed to clarify intervention effects [41]. Given that the intervention effect on task crafting was non-significant at post-intervention (*d* = 0.15; 95 % CI −0.15 to 0.44) but became significant at one-month follow-up (*d* = 0.32; 95 % CI 0.02 to 0.62), longer follow-up will be needed to detect the effect of the program on outcomes such as task crafting or relational crafting in future studies.

Fifth, the current study did not assess job or personal resources, which were predictors of work engagement in Job Demands-Resources model [17], because the main purpose of the study was to investigate the effectiveness of our program on work engagement preliminarily. Accordingly, the further study would be needed to investigate the effect on job and personal resources. It would clarify the underlying mechanisms whether job crafting would improve work engagement through job and personal resources.

## Conclusion

This single group pre- and post-intervention study demonstrated that the newly developed job crafting program was effective in improving work engagement, as well as in improving job crafting and decreasing psychological distress, among Japanese managers.
